# Commentary: Immunochemical Markers of the Amyloid Cascade in the Hippocampus in Motor Neuron Diseases

**DOI:** 10.3389/fneur.2017.00105

**Published:** 2017-03-20

**Authors:** Ian Paul Johnson, Cintia Roodveldt

**Affiliations:** ^1^Anatomy and Pathology, Adelaide Medical School, University of Adelaide, Adelaide, SA, Australia; ^2^Andalusian Center for Molecular Biology and Regenerative Medicine-CABIMER, Unversity of Seville-CSIC-University Pablo de Olavide, Seville, Spain

**Keywords:** neurodegenerative diseases, aging, common features, amyloid cascade, translational medical research

In case we needed reminding, age-related neurodegenerative diseases such as Alzheimer’s disease (AD), Parkinson’s disease (PD), and motor neurone disease (MND) have two factors in common: (i) advancing age as the single biggest risk factor and (ii) the fact that they are characterized by neuronal degeneration. A recent article (1) encourages us to focus on some of the similarities of these diseases by demonstrating that features characteristically associated with AD are also commonly found in MND.

In their study (1), the group measured key biomarkers of the amyloid cascade [amyloid precursor protein (APP), transactive response DNA-binding protein 43 (TDP-43), phosphorylated TDP-43 (pho-TDP43), amyloid-beta peptide (Aβ), and amyloid precursor protein-binding protein family B (Fe 65)] immunohistochemically in postmortem samples of the hippocampus of amyotrophic lateral sclerosis (ALS) and ALS–frontotemporal dementia patients. Compared to controls, they report increased levels of APP and Aβ peptide in MND patients; the latter change also correlating with cytoplasmic pho-TDP-43 expression. In addition, they found decreased Fe65 expression and increased expression of pho-tau. Interestingly, these molecular alterations were similar for both ALS and ALS–FTD, albeit more pronounced in the latter group. This indicates that the “amyloid cascade,” resulting in the accumulation of amyloid β, is activated in the hippocampus of patients with ALS and ALS–FTD, and that such activation correlates with alterations in TDP-43.

This is an important finding, because it adds to the growing body of evidence that age-related neurodegenerative diseases, rather than being discrete entities, may in fact be different points on a continuum, and the corollary of this is that they may all have similar underlying mechanisms. Indeed, a brief survey of three age-related neurodegenerative diseases (AD, PD, and MND) reveals that there is much overlap in the features associated with these conditions (Table 1), and that they have more in common than anything else (Figure 1).

**Table 1:** **Typical and associated features of Alzheimer’s disease (AD), Parkinson’s disease (PD), and motor neurone disease (MND)**.

Condition	Typical features	Associated features
AD	**1.** Aβ deposition (amyloid plaques), intraneuronal aggregation of pho-tau	**9.** Movement disorder
	**2.** Increased amyloid precursor protein (APP), accumulation of Aβ and pho-tau	
	**3.** Cholinergic dysfunction	
	**4.** Cognitive changes	
	**5.** Sustained neuroinflammation	
	**6.** Accumulation of ubiquitinated proteins	

PD	**5.** Sustained neuroinflammation	**3.** Cholinergic dysfunction
	**6.** Accumulation of ubiquitinated proteins	
	**7a.** Intracellular accumulation of α-synuclein amyloid-like fibrils (Lewy bodies)	**4.** Cognitive changes
	**8.** Nigral dopamine loss	
	**9.** Movement disorder	

MND	**5.** Sustained neuroinflammation	**2.** Increased APP, accumulation of Aβ and pho-tau
	**6.** Accumulation of ubiquitinated proteins	
	**9.** Movement disorder	**4.** Cognitive changes
	**10.** C9ORF72 mutation	**7b.** Intracellular α-synuclein/SOD1 co-aggregation in amyotrophic lateral sclerosis
	**11.** Intracellular accumulation of aggregated proteins (TDP-43, FUS, and SOD1)	

*Numbers refer to the following references: ***(1)*** Hinz and Geschwind (2); Aday and Sze (3); ***(2)*** Vilemagne et al. (4); Gomez-Pinedo et al. (1); ***(3)*** Ballinger et al. (5); Park et al. (6); ***(4)*** Brandt et al. (7); ***(5)*** Chen et al. (8); Amor et al. (9); ***(6)*** Atkin and Paulson (10); Jansen et al. (11); ***(7a)*** Uchihara and Giasson (12); ***(7b)*** Helferich et al. (13); ***(8)*** Kalia and Lang (14); ***(9)*** Brandt et al. (7); ***(10)*** Ticozzi et al. (15); ***(11)*** Guerrero et al. (16)*.

**Figure 1:**
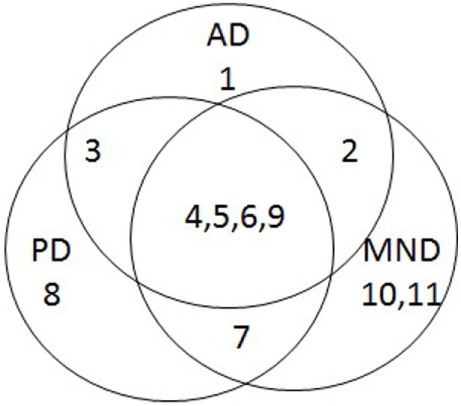
**Venn diagram summarizing overlap of features associated with Alzheimer’s disease (AD), Parkinson’s disease (PD), and motor neurone disease (MND)**.

The paper by Gomez-Pinedo et al. (1) is a timely reminder that researchers should perhaps not get tied up with the details of these individual diseases that are so important for differential diagnoses. Instead, those seeking to illuminate basic underlying mechanisms might do well to pool data for neurodegenerative diseases in the hope that it will point them in the right direction. After all, these conditions have so far defied effective treatment or cure.

## Author Contributions

IJ wrote the initial draft. CR revised the initial draft and contributed with further writing. Both collected data from literature and revised the final manuscript.

## Conflict of Interest Statement

The authors declare that the research was conducted in the absence of any commercial or financial relationships that could be construed as a potential conflict of interest.
